# Integrative analysis of multiple gene expression profiles with quality-adjusted effect size models

**DOI:** 10.1186/1471-2105-6-128

**Published:** 2005-05-27

**Authors:** Pingzhao Hu, Celia MT Greenwood, Joseph Beyene

**Affiliations:** 1 The Hospital for Sick Children Research Institute, 555 University Ave., Toronto, ON, M5G 1X8, Canada; 2 Department of Public Health Sciences, University of Toronto, 1 King's College Circle, Toronto, ON, M5S 1A8, Canada

## Abstract

**Background:**

With the explosion of microarray studies, an enormous amount of data is being produced. Systematic integration of gene expression data from different sources increases statistical power of detecting differentially expressed genes and allows assessment of heterogeneity. The challenge, however, is in designing and implementing efficient analytic methodologies for combination of data generated by different research groups.

**Results:**

We extended traditional effect size models to combine information from different microarray datasets by incorporating a quality measure for each gene in each study into the effect size estimation. We illustrated our method by integrating two datasets generated using different Affymetrix oligonucleotide types. Our results indicate that the proposed quality-adjusted weighting strategy for modelling inter-study variation of gene expression profiles not only increases consistency and decreases heterogeneous results between these two datasets, but also identifies many more differentially expressed genes than methods proposed previously.

**Conclusion:**

Data integration and synthesis is becoming increasingly important. We live in a high-throughput era where technologies constantly change leaving behind a trail of data with different forms, shapes and sizes. Statistical and computational methodologies are therefore critical for extracting the most out of these related but not identical sources of data.

## Background

The introduction of DNA microarray technology has enabled investigators to screen thousands of genes simultaneously. One of the main goals of these studies is to identify differentially expressed genes between two biological conditions. For example, many studies [1-4] have been performed in prostate cancer research to find candidate markers. Since laboratory protocols, microarray platforms and analysis techniques used in these studies were not identical, it is difficult to make a comparison among the results obtained from them. However, systematic integration of gene expression data from different sources increases statistical power of detecting differentially expressed genes and allows assessment of heterogeneity. Meta-analysis is a classical statistical methodology for combining results from different studies addressing the same scientific questions, and it is becoming particularly popular in the area of medical and epidemiological research [5]. Meta-analysis methods have recently been applied to the analysis of microarray data [6-11].

Rhodes et al. [6] focused on combining p-values for each gene from the individual studies to estimate an overall p-value for each gene across all studies. Their method has been applied to four prostate cancer microarray datasets, two of which are cDNA microarray data and the remainder Affymetrix microarray data. Samples in each data set were taken from prostate cancer cases, but were analyzed with different platforms. Differential expression was assessed independently for each gene in each dataset. Since the method chosen to combine results across studies was based on the statistical confidence measure, the p-value, not on the expression level, this strategy avoids direct comparisons of data sets and related cross-platform normalization issues. Choi et al. [7] focused on integrating effect size estimates to obtain an overall estimate of the average effect size. Effect size is used to measure the magnitude of treatment effect in a given study. Using the same datasets as those used by Rhodes et al. [6], they demonstrated that their method can lead to the discovery of small but consistent expression changes with increased sensitivity and reliability.

Parmigiani et al. [9] developed a correlation-based method for assessing reproducibility of gene expression studies with application to lung cancer. They demonstrated that their method can improve correlation across the various studies. Jiang et al. [10] used a distribution transformation method to integrate two lung cancer studies and proposed a gene shaving-based classification approach to identify a small list of differentially expressed genes between lung cancer and normal patients. They noted that many of the selected genes have been experimentally validated.

Although some of the above studies (for example Rhodes et al. [6], Choi et al. [7], Parmigiani et al. [9]) demonstrated the utility of integrating cDNA and Affymetrix microarray data, other investigators argued against this approach. Kuo et al. [12] compared Affymetrix and spotted cDNA gene expression measurements based on 60 cell lines from the National Cancer Institute. They found low correlation between the actual gene measurements from the two technologies, and concluded that "data from spotted cDNA microarrays could not be directly combined with data from synthesized oligonucleotide arrays." Moreover, they concluded that it was unlikely that the two types of data could be transformed or normalized into a common standardized index. Jarvinen et al. [13] determined the level of concordance between microarray platforms by analyzing breast cancer cell lines with in situ synthesized oligonucleotide arrays, commercial cDNA microarrays and custom-made cDNA microarrays. Their results demonstrated that data from different microarray platforms are variable to the extent that direct integration of data from different platforms may be complicated and unreliable.

In classical meta-analysis, quality measures have often been used when combining results across studies. It has been argued that studies of a higher quality will give more accurate estimates of the true parameter of interest, and therefore studies of high quality should receive a higher weight in the analysis summarizing across studies [14]. The Affymetrix microarray technology has been used worldwide. Its success has been demonstrated by numerous publications in scientific journals. However, it is well-known that only part (approximately 40–50%) of the whole genome is expressed in any specific tissue type, so there are many genes showing low expression and random variability across samples. Furthermore, some genes will be measured less precisely by some technologies, or studies, than others.

Therefore, our ability to develop powerful statistical methods for efficiently integrating and weighting information from related genomic experiments will be critical in the success of the massive investment made on genomic studies. The focus of this paper is to design and implement a quality measure appropriate for Affymetrix microarray data. Using our quality measure, we weighted the importance of each gene in each experiment and incorporated our quality measure into the effect size model proposed by Choi et al. [7] to model inter-study variation of gene expression profiles. We believe that applying this approach can lead to a more accurate description of expression patterns than Choi et al's method [7].

## Results

**Figure 1:**
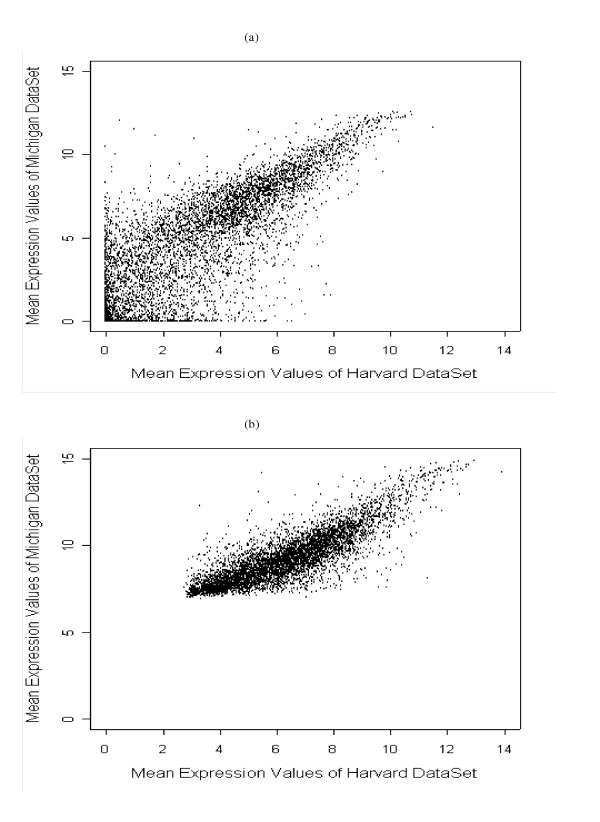
Scatter plots of (a) quality-weighted and (b) quality-unweighted mean expression values for the 6124 common probe sets in the Harvard and Michigan datasets based on normal (healthy) samples.

We used two data sets consisting of gene expression profiles in lung cancer and normal subjects. These datasets were collected using different chip types of the Affymetrix oligonucleotide microarrays and were conducted by two research groups, one from Harvard and the other from Michigan (see Methods section for details). A list of 6124 common probe sets found in the two datasets was used for data analysis in this study [10]. We developed a quality weight for each gene in each study by modeling the log of the detection p-values with an exponential distribution, and then summarizing across arrays and groups within each study (see Methods). In order to visualize the effect of the proposed quality weighting, we calculated the mean expression value of each probe set across all normal samples, where the expression variation presumably is less heterogeneous than among the cancer samples. The mean expression value of a probe set in a study is the estimated average of the probe set's intensity values across all normal samples in the study. Figure 1 shows the scatter plot of the average expression values of the probe sets in the Harvard dataset plotted against that of the Michigan dataset: (a) weighted by the quality score (see Methods section for our definition of the quality score), and (b) un-weighted. This plot is intended to be illustrative only – our algorithm weights the test statistics, rather than the gene expression measures. In Figure 1(a), it can be seen, as expected, that many of the genes with low levels of expression are associated with low quality weights.

**Figure 2:**
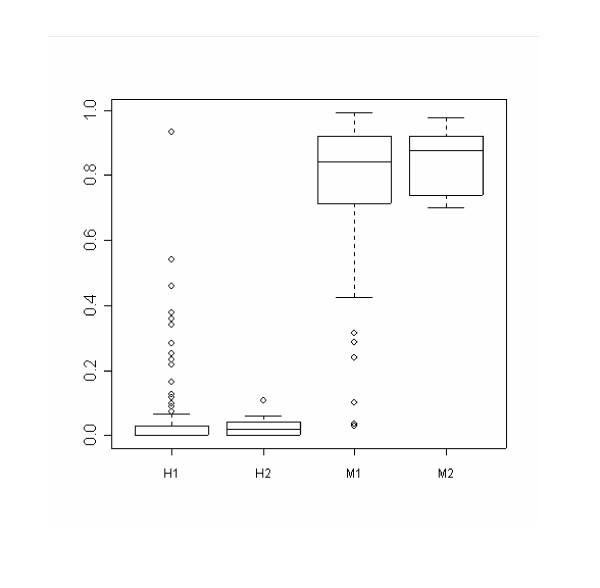
Detection p-values for a sample probe set (38249_at). H1 and H2 denote the detection p-values in normal and lung cancer groups, respectively, for the Harvard study; whereas M1 and M2 denote the detection p-values in normal and lung cancer groups, respectively, for the Michigan study.

We show examples of quality scores for selected probe sets in Table 1. The two datasets may have very different detection p-value distributions, which are reflected in the quality scores. Figure 2 shows a box plot of the detection p-values for one probe set from Table 1. When the two datasets give small p-values (e.g., last line in Table 1), the minimum p-value may be much smaller in one dataset than another. Both, however, will give high quality scores with an appropriate choice of the sensitivity parameter *s *that adjusts how the quality measure interprets the detection p-values.

**Figure 3:**
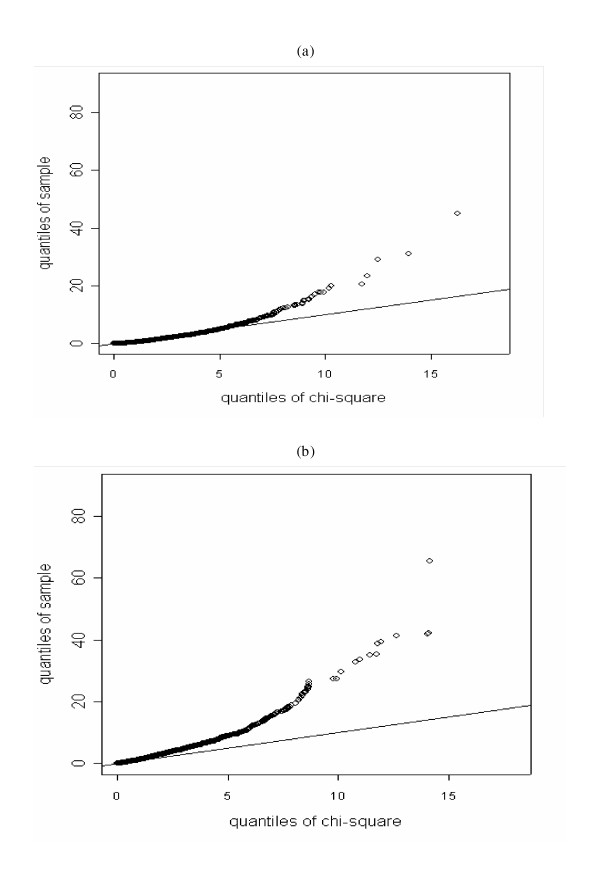
Quantile – Quantile plots of the observed versus the expected Q statistic: (a) with quality adjustment, and (b) without quality adjustment.

Figure 3 shows the adjusted and unadjusted quantile – quantile (Q-Q) plots of the observed vs. expected Q values. Q is the test statistic we used for assessing heterogeneity, and is described in detail later in the Methods section. In the adjusted Q-Q plot, the quality score was used as a weight in the computation of Q while it was not considered in the unadjusted Q-Q plot. From these graphs, we can see that the quantiles of the observed Q values are far from the expected quantiles of a  distribution, suggesting that these two datasets generated heterogeneous results beyond random sampling errors. Therefore, we applied the random effect model in this study. The quantiles of the Q statistic were closer to the quantiles of the expected chi-square distribution when quality-adjustment was considered (Figure 3(a)) than when it is was not (Figure 3(b)). The variance for the unadjusted *Q *values was 9.45, but it was reduced to 3.31 when quality adjustment was used. This result suggests that the incorporation of the adjusted quality measure into effect size estimation can increase consistency and decrease heterogeneity between these two datasets.

**Figure 4:**
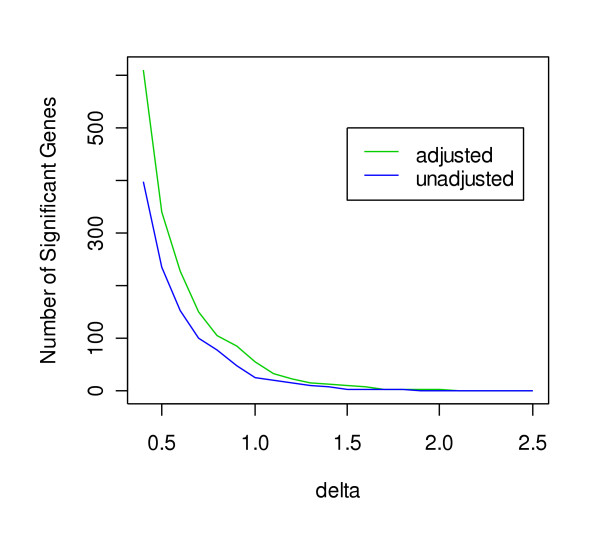
Relationship between number of significantly expressed genes and different delta levels, obtained from fitting the random effects model.

To identify a list of potentially "significant" genes, we adapted the false discovery rate (FDR) algorithm implemented in [30]. We first calculated the adjusted z statistics for all genes based on random-effects model (REM). Genes were then ranked by the magnitude of their z statistic values. A permutation-based approach was used to obtain the corresponding expected ordered z statistic. The potentially "significant" genes are genes with a distance between the ordered z statistic from the observed data and that of the permuted data exceeding a given threshold (delta). Figure 4 shows the relationship between the number of significantly differentially expressed genes and different delta levels. As we see in this figure, the quality-adjusted REM can identify many more significant genes than the quality-unadjusted REM model at any fixed level of delta.

We calculated the estimated FDR for each given delta. As expected, the number of genes called significant increased as the value of delta decreased, but at the cost of an increasing FDR. The estimated FDR was zero down to a delta of 0.6, where 228 genes were called significant in the quality-adjusted model and 153 genes in the quality-unadjusted model. In order to get a manageable gene list, we arbitrarily set delta at 1.1. At this delta level, we selected 29 differentially expressed genes (representing 32 probe sets) at a FDR of 0% when the quality weight was incorporated into the estimation of the effect size. However, when the quality measure was not used (Choi et al's method [7]), we only selected 20 differentially expressed genes (representing 21 probe sets) at a FDR of 0%. All the 20 genes were also in the top of the list of the 29 genes identified with the quality-weights. Tables 2 and 3 show the selected differentially expressed genes between normal and lung adenocarcinoma patient samples using the quality-adjusted and quality-unadjusted models, respectively, with genes ordered based on their z statistic values [see Additional files 1 and 2]. As can be seen in Tables 2 and 3, 4 of the 9 genes that were selected by our method, but not by Choi et al's method, have also been identified by several other groups including Jiang et al. [10], Beer et al. [15] and Bhattacharjee et al. [16]. In particular, some of these 4 genes, such as TEK and TGFBR2, have been experimentally validated (shown in Table 3 of Jiang et al.[10]). For a cutoff of an adjusted z value of 1.96 (corresponding to a 5% level of significance), the quality-adjusted model identified 9 significantly expressed genes while the quality-unadjusted model identified only 2 of the 9 significantly differentially expressed genes. All these results suggest that our proposed method may have increased sensitivity to detect more differentially expressed and biologically relevant genes than Choi et al's method [7].

We compared genes identified with our method with genes identified by Jiang et al. [10], Beer et al. [15] and Bhattacharjee et al. [16], sixteen of the 29 genes identified by our proposed model were also detected in at least one of these studies. In particular, we observed that 6 of the 29 genes were consistently identified in the other three studies. There were 13 of the 29 genes that were uniquely identified in our study. Some of these are plausible candidates for lung adenocarcinoma. For example, Walker et al. [17] found that G protein-coupled receptor kinase 5 (GRK5) is a key gene regulating airway response, that may have implications in obstructive airway diseases.

## Discussion

In this study we proposed a measure to quantify Affymetrix gene chip data quality for each gene in each study. The quality index measures the performance of each probe set to detect its intended target. Furthermore, we extended a traditional effect size model by using the quality index as a weight for combining information from different chip types of Affymetrix microarrays, and incorporating this weight into a random-effects meta-analysis model. We illustrated the advantages of our proposed methods using the Harvard and Michigan gene expression datasets used in [18]. This approach of using the detection p-values to weight the gene expression estimates can be applied in a more general context and to other microarray meta-analysis methods, such as that of Rhodes et al [6].

The assumption of a group-specific exponential distribution for the negative log detection p-values is a very rough approximation. The true distribution is probably closer to the two-parameter log-beta distribution. However, due to the fact that there are only 16–20 probe pairs per probe set used in the Wilcoxon test statistic for the detection p-values, the p-values follow a somewhat discrete distribution. In particular for highly-expressed genes, the p-values for all samples in a group may have the same near-zero value. Estimation of two-parameters is therefore impossible. It would be worth investigating the sensitivity of the performance of this quality-weighting approach to the distributional assumption.

The performance of the weighting also depends on the sensitivity parameter s. In this study, a high *s *was needed since a lower cutoff gave almost all genes extremely low quality scores. This is a consequence of the observed detection p-value distributions. These data were created in 2001, and since that time the quality of the oligonucleotide array technology has improved with more specific probe selections and optimized experimental conditions. For expression data obtained today, smaller values of s would be more appropriate. For example, in an analysis of a dataset generated more recently using improved oligonucleotide technology (not shown), using *s *= 0.05 gave good performance.

The discrepancy between the calculated and expected values for the Q test of heterogeneity was smaller when our quality measure was incorporated than when it was not, as suggested by the quantile-quantile plots, but overall there still remains some variability that has not been fully captured by the weight function, emphasizing the need for a more elaborate weighting strategy and sensitivity analyses.

From a biological point of view, lung adenocarcinoma may be heterogeneous originating from different causes [19] and methods like cluster analysis in which subtypes of relatively homogeneous groups of disorders can be identified might be useful. The focus of our paper was to introduce a methodology that can be used to integrate data, and as such the grouping of the samples as "diseased" versus "normal" was primarily used to identify genes that discriminate the two groups in a broader sense than at a higher resolution in which sub types could be identified.

Choi et al. [7] have also provided a brief Bayesian interpretation of their effect size model to integrate information from multiple microarray studies. They argued that a Bayesian approach could offer a more flexible and robust modeling strategy. We did not consider this issue in this study. It will be interesting to investigate how our proposed quality measure could be incorporated into their Bayesian framework in the future.

## Methods

### Data source and preprocessing

We selected two Affymetrix microarray data sets from the 4 lung cancer data sets provided by the organizers of the Critical Assessment of Microarray Data Analysis (CAMDA) conference [18]. These datasets were collected using different versions of the Affymetrix oligonucleotide microarrays and were conducted by two research groups, one from Harvard and the other from Michigan. The Michigan study [15] used the HuGeneFL Affymetrix chip, containing 7,129 probe sets, each with 20 probe pairs. This study included 86 lung adenocarcinoma patient samples and 10 normal samples. The Harvard study [16] used the HG_U95Av2 chip with 12,625 probe sets, each with 16 probe pairs. This study included 17 normal and 127 lung adenocarcinoma patient samples. We use the word "sample" interchangeably with "array". The main objectives addressed in these two studies were to identify differentially expressed genes related to lung adenocarcinoma, and genes whose expression was related to patient survival. Our interest is in the former. We developed a new method for identifying genes that are differentially expressed between the cancer and normal samples, by modelling the effect size and integrating information from the Harvard and Michigan studies.

We converted the probe level data to a single expression measure for each probe set using the robust multi-array average (RMA) algorithm [20], which provides higher specificity and sensitivity in detecting differentially expressed genes. We used 6124 common probe sets in these two studies for the data analysis in our study. These probe sets were selected based on a sequence-based probe matching method, which is believed to produce more consistent results when comparing similar biological data sets obtained by different microarray platforms [10,21]. The detailed procedure used to select these probe sets can be found in the Data processing Section of [10].

### Quality measure for Affymetrix Genechip data

Affymetrix genechip microarrays are used to monitor gene expression for thousands of transcripts. Each transcript is represented as a probe set, and a probe set is made up of probe pairs comprised of Perfect Match (PM) and Mismatch (MM) probe cells. The level of expression of a gene product is estimated using the intensities of each probe pair in a probe set. Therefore, the probe-specific variability in a probe set can be used as a measure of the performance of that probe set. The detection algorithm proposed by [22] generates a detection p-value, which represents the probability that the probe set (gene) expression is above zero (i.e., turned on), and measured reliably and consistently. A lower p-value is considered as a useful indicator that the measured gene expression is valid and reliable [22]. Specifically, the p-value is based on testing whether the probe-specific differences (PM-MM) are almost always positive. We used the detection p-values to define quality measures for probe sets, summarizing across the arrays and experiments. We realize that some genes may be truly "off" under some experimental conditions and hence a large detection p-value may be providing useful information. However, if a gene is "off" under all experimental conditions, we argue that analysis of this gene contributes little to understanding of the experiment.

For any gene, let *pvalue *denote its detection p-value and *x*_lg _denote - log(*pvalue*_lg_) for sample *l *in group *g*. We assume that each study compares G groups, where there are *n*_*g *_samples in group g, and g = 1, 2, ..., G. For example, in the lung cancer data, G = 2, since adenocarcinoma samples are compared to controls. It is well known that p-values follow a uniform distribution when there is no signal, and therefore, we expect *x*_lg _to follow an exponential distribution with mean λ = 1 if the gene is not expressed. In order to develop a single quality measure for each gene across all samples in one study, we use this relationship with the exponential distribution to motivate a quality measure. We assumed that the detection p-value for a single gene of sample *l *in group *g *follows the distribution

*x*_lg _= - log (*pvalue*_lg_) ~ *Exponential *(λ_*g*_),

where different distributions of expression can be expected in each group g. It should be noted that we are modeling the p-value of one gene, across the samples in a group. This is different from the approaches of Allison et al. [23] and Pounds et al. [24] who modeled the distribution of p-values across genes. Although the true distribution of the *x*_lg _may not be exponential, this assumption leads to a simple model where the one parameter can be estimated by a closed-form expression. Hence, the parameter λ_*g *_for each gene, study and group *g *can be estimated by:



where  is the usual sample mean. This is a maximum likelihood estimator (MLE) with well-known asymptotic optimality properties [25].

To combine across the groups, we assumed a sensitivity parameter *s*. It is defined as the probability that a representative probe set in a particular treatment group shows a detectable signal, assuming that the relevant distribution (exponential or beta) holds. It can be thought of as the equivalent to a detection p-value defined for a whole group rather than for one array, and an appropriate value for *s *should be chosen with this interpretation in mind. For example, the default settings by Affymetrix software for the detection calls are p = 0.06 for a "Marginal" call, and p = 0.04 for a "Present" call, although these can be altered by the user. In practice, the appropriate choice of *s *may depend on the signal-detection capability of particular technologies. We recommend plotting the distribution of quality scores for different choices of *s*, and choosing a value that clearly distinguishes genes of low quality (scores near zero) from high quality genes (scores near 1).

The sensitivity parameter *s *is a chosen cutoff, so that genes that are "off" or poorly measured across all experimental conditions will have *pvalue *≥ *s*, or in other words, . Therefore, we can define a quality measure across the groups, for each gene and each study as:



The choice of the maximum gives more weight to genes measured with high quality in at least one group, thereby allowing a gene that is "off" in one condition and "on" under another condition to provide useful information in the analysis. We treat this quality score as a weight for each gene in the subsequent analysis.

### Modelling effect size with quality-adjusted weights

In order to simplify the discussion, we consider two groups, treatment (*t*) and control (*c*) groups, in study *i *= 1,2,...,*k*. Let *n*_*it *_and *n*_*ic *_denote the number of treatment and control samples in study *i*, respectively. For each gene, let μ denote its overall mean effect size, a measure of the average differential expression for that gene, and let *y*_*i *_denote the observed effect size for study *i*. We modeled effect size using the hierarchical model:



Where τ^2 ^is the between-study variability and  is the within-study variance, measuring the sampling error for the *i*^*th *^study. Choi et al. [7] used the standardized mean difference as a measure of the observed treatment difference *y*_*i*_. This well-known estimator of treatment difference found in Hedges and Olkin's [26] work is



where  and  are the average gene expression values in the treatment and control groups of study *i*, respectively, and  is the pooled standard deviation.

For a study with *n *samples, an approximately unbiased estimator of  is given by [26]. The estimated variance  of the unbiased effect size is given by [27]



In a fixed-effects model (FEM), the error of the observed effect sizes is fully assigned to sampling error only, ignoring the between study variance, so τ^2 ^= 0 and *y*_*i *_~ *N *(μ, ). On the other hand, a random-effects model (REM) considers that each study estimates a different treatment effect θ_*i*_. These parameters are drawn from a normal distributionθ_*i *_~ *N*(μ, τ^2^).

To assess whether FEM or REM is most appropriate, we tested the hypothesis τ = 0 using the following test statistic, which is a modification of Cochran's test statistic [28] that incorporates our quality measure for each study



where  and



 is the weighted least squares estimator that ignores between study variations. Under the null hypothesis of τ = 0, this statistic follows a  distribution. We followed Choi's approach [7] to draw quantile-quantile plots of *Q *to assess whether a FEM or REM model is appropriate. If the null hypothesis of τ = 0 is rejected, we estimate τ based on the method developed by DerSimonian and Laird [29]



Therefore, we can estimate μ that corresponds to a random effects model by



where . Under the REM,



The *z *statistics to test for treatment effect under REM is



However, when there are only a small number of arrays in each group, the estimates of standard error *s *for each gene can be very variable. Some genes might by chance have very small standard errors, and therefore appear highly significant. To address this problem, different approaches have been developed for "smoothing" the variance estimates by borrowing information from the ensemble of genes. This can assist in inference about each gene individually. For example, Tusher et al. [30], Efron et al. [31] and Broberg [32] used t-statistics where an offset was added to standard deviation while Smyth [33] proposed a t-statistic with a Bayesian adjustment to the denominator. We took the offset *s*_0 _as the quantile of the gene-wise standard errors that minimizes the coefficient of variation of the z statistics [30]. Therefore, we can calculate the adjusted *z *statistics (used in this study) to test for treatment effect under REM as



The adjusted *z *statistics for FEM is the same as that for REM except that τ^2 ^= 0. Note that all these expressions refer to a single gene. The adjustment for computing z statistic was also used by Garrett-Mayer et al. [34].

### Assessment of differentially expressed genes

We performed a multiple testing procedure, as described by Dudoit et al. [35], to evaluate statistical significance for differentially expressed genes in the combined studies. The false discovery rate (FDR) [36] has become a popular error measure for this purpose. Tusher et al. [30] developed a permutation-based method to calculate FDR for evaluating differentially expressed genes. We adapt their approach to our meta-analytic framework as follows:

1. For each gene *j, j *= 1,2,..., *J*, in the original data, compute the adjusted z statistic *Z*_1_,...,*Z*_*J *_based on the meta-analysis procedure described in the previous section

2. Order these z statistic values to obtain *Z*_(1) _≤,..., ≤ *Z*_(*J*)_

3. Create B random permutations within both studies. For each permutation *b *= 1,2,... *B*, produce the adjusted z statistic *Z*_1,*b*_,..., *Z*_*J*,*b *_for gene *j *= 1,2,..., *J*

4. Compute expected order z statistics:



5. The potentially "significant" genes are those that have a distance between *Z*_(*j*) _and , greater than a given threshold delta (Δ). Therefore, we can find the smallest positive *Z*_(*j*) _such that , say *t*_1_. Similarly, we can find the largest negative *Z*_(*j*)_, say *t*_2_

6. The estimated FDR for the selected significant genes at the given delta (Δ) is given by:



## Authors' contributions

JB initiated, designed and managed the study. CG proposed the quality measure method and participated in designing and managing the study. PH conducted data analysis and drafted the manuscript. JB and CG revised the manuscript. All authors read and approved the final manuscript.

## Supplementary Material

Additional File 1 Selected differentially expressed genes between normal and lung adenocarcinoma patient samples (Genes selected based on *quality-adjusted model *with delta = 1.1) Click here for file

Additional File 2 Selected differentially expressed genes between normal and lung adenocarcinoma patient samples (Genes selected based on *quality-unadjusted model *with delta = 1.1) Click here for file

## Figures and Tables

**Table 1:** Quality scores for selected probe sets at a sensitivity parameter s = 0.2

	Quality Score		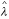	
	
Probe Set	Harvard Study	Michigan Study	Harvard Study	Michigan Study
38249_at	0.663	0.001	0.255	4.050
32180_s_at	0.263	0.732	0.829	0.194
37174_at	0.001	0.495	4.050	0.437
32318_s_at	0.795	0.795	0.142	0.142
